# Cross-platform comparison of SYBR^® ^Green real-time PCR with TaqMan PCR, microarrays and other gene expression measurement technologies evaluated in the MicroArray Quality Control (MAQC) study

**DOI:** 10.1186/1471-2164-9-328

**Published:** 2008-07-11

**Authors:** Emi Arikawa, Yanyang Sun, Jie Wang, Qiong Zhou, Baitang Ning, Stacey L Dial, Lei Guo, Jingping Yang

**Affiliations:** 1 SuperArray Bioscience Corporation, Frederick, MD 21704, USA; 2 National Center for Toxicological Research, US Food and Drug Administration, Jefferson, AR 72079, USA

## Abstract

**Background:**

The MicroArray Quality Control (MAQC) project evaluated the inter- and intra-platform reproducibility of seven microarray platforms and three quantitative gene expression assays in profiling the expression of two commercially available Reference RNA samples (***Nat Biotechnol ***24:1115-22, 2006). The tested microarrays were the platforms from Affymetrix, Agilent Technologies, Applied Biosystems, GE Healthcare, Illumina, Eppendorf and the National Cancer Institute, and quantitative gene expression assays included TaqMan^® ^Gene Expression PCR Assay, Standardized (Sta) *RT*-PCR™ and QuantiGene^®^. The data showed great consistency in gene expression measurements across different microarray platforms, different technologies and test sites. However, SYBR^® ^Green real-time PCR, another common technique utilized by half of all real-time PCR users for gene expression measurement, was not addressed in the MAQC study. In the present study, we compared the performance of SYBR Green PCR with TaqMan PCR, microarrays and other quantitative technologies using the same two Reference RNA samples as the MAQC project. We assessed SYBR Green real-time PCR using commercially available RT^2 ^Profiler™ PCR Arrays from SuperArray, containing primer pairs that have been experimentally validated to ensure gene-specificity and high amplification efficiency.

**Results:**

The SYBR Green PCR Arrays exhibit good reproducibility among different users, PCR instruments and test sites. In addition, the SYBR Green PCR Arrays have the highest concordance with TaqMan PCR, and a high level of concordance with other quantitative methods and microarrays that were evaluated in this study in terms of fold-change correlation and overlap of lists of differentially expressed genes.

**Conclusion:**

These data demonstrate that SYBR Green real-time PCR delivers highly comparable results in gene expression measurement with TaqMan PCR and other high-density microarrays.

## Background

Gene expression research is a rapidly evolving field with recent advances in technologies aimed at multi-gene expression profiling and high throughput screening. Technologies like high-density DNA microarrays enable one to perform parallel gene expression profiling in the scale of tens of thousands of genes in a single experiment [1,2]. Quantitative real-time-PCR, though lacking the scale of microarrays, is a rapid, sensitive and less complex method for gene expression analysis and offers an alternative approach for parallel profiling of multiple targets as well as a time-saving means to validate microarray results.

With many different technologies available for gene expression measurement, the need to compare the results obtained from different platforms and technologies and thus the reliability and biological significance of those results becomes evident. Moreover, concerns regarding the reliability and consistency of the microarray technology from different suppliers, different test sites and when using different methods for data processing and normalization have been raised [3-7]. To address those concerns, scientists from the US Food and Drug Administration (FDA) established the MicroArray Quality Control (MAQC) consortium to evaluate the performance of several microarray platforms as well as three quantitative gene expression assays [8-13]. The microarray platforms were from Affymetrix (AFX), Agilent Techonologies (one-color protocol (AG1) or two-color protocol (AGL)), Applied Biosystems (ABI), GE Healthcare (GEH), Illumina (ILM), Eppendorf (EPP) and the National Cancer Institute (NCI), and the three quantitative assays were TaqMan^® ^Gene Expression Assay (Applied Biosystems, Foster City, CA), Standardized (Sta) *RT*-PCR™ (Gene Express, Inc., Toledo, OH) and QuantiGene^® ^(Panomics, Inc., Fremont, CA).

Reports from the Phase 1 study of the MAQC project, which profiled two standardized reference RNA samples, contain important findings on the performance of different expression measurement technologies and give insights into the level of cross-platform comparability among different technologies [8-13]. The comprehensive data sets generated from this MAQC effort showed that great inter-site and cross-platform consistency can be achieved among different technologies [8,12]. Importantly, the selection criteria used to define differentially expressed genes has a substantial impact on the overlap of the resulting gene lists, with gene lists generated by fold change ranking being more reproducible than those obtained by *t*-test *P *value ranking. With these findings, the MAQC Consortium recommends fold change ranking using a nonstringent P-value cutoff for gene selection. Another important goal attained by this project is to generate a thoroughly characterized reference data set against which new modifications in the existing microarray platforms and other expression measurement technologies can be compared and validated, and laboratory performance can be assessed. This was accomplished by providing the community with two commercially available high-quality human reference RNA samples that can be used as a tool for calibration and quality control as well as for performance assessment and validation of assays. The two RNA samples used in the MAQC project were the Stratagene Universal Human Reference RNA (comprised of RNA from ten different cell lines) and the Ambion Human Brain Reference RNA. Extremely large lots of these two reference RNAs were produced under stringent quality-control procedures. This has allowed researchers to assess the performance of their assays over time using the same RNA samples from identical manufacturing lots and to compare their results with the MAQC data set.

The platforms evaluated in the MAQC project can be categorized into either hybridization-based or PCR-based technologies. Different platforms of microarray and QuantiGene assays belong to hybridization-based technology. For microarrays, RNA samples are labeled with a tag (biotin or a fluorophore) followed by hybridization to immobilized gene-specific probes and fluorophore-based detection. QuantiGene is a sandwich nucleic acid hybridization system that detects RNA directly [14]. Targets are captured through joint hybridization of multiple probes, and the complex is detected by signal amplification through a branched DNA amplifier and chemiluminescence signal production. TaqMan Gene Expression Assays and Sta*RT*-PCR are PCR-based techniques. Sta*RT*-PCR is a competitive end-point PCR-based assay. A standardized mixture of internal standard (SMIS) competitive templates is added to the reverse transcribed products prior to PCR. The individual endpoint Sta*RT*-PCR products are then separated by size and quantified by high-throughput microfluidic electrophoresis [15]. The TaqMan assay is based on real-time PCR using a fluorescent dye to monitor the amplification of target genes by DNA polymerase. It employs a target-specific, dual-labeled, fluorogenic hybridization probe with a quencher on the 3' end to be hydrolyzed by the 5' to 3' exonuclease activity of Taq polymerase during the extension step [16].

Real-time PCR is widely considered the gold standard for gene expression measurement due to its high assay specificity, high detection sensitivity and wide linear dynamic range. In addition to the TaqMan assay, the SYBR^® ^Green PCR assay is another commonly used real-time PCR technique which is employed by half of all real-time PCR users [17]. Despite its widespread use, this technique was surprisingly not included as part of the MAQC project. SYBR Green PCR is widely used because of the ease in designing the assays and its relatively low setup and running costs. Unlike TaqMan fluorescent probes, SYBR Green dye intercalates into double-stranded DNA to monitor the amplification of target gene specifically initiated by gene-specific primers. One drawback of SYBR Green assays, however, is that the dye is non-specific and can generate false positive signals if non-specific products or primer-dimers are present in the assay. Those problems can be addressed by carefully designing the primers and validating the PCR products with dissociation curve analysis immediately after PCR. In addition, other approaches have been practiced to further increase the specificity of SYBR Green detection, such as a "hot start" strategy using a DNA polymerase that requires heat activation, or acquisition of fluorescence signals at a temperature slightly below the melting temperature of the desired amplicon but above which nonspecific primer-dimer related products will denature and produce minimal signals [18,19].

In the present study, we have evaluated the performance of SuperArray's SYBR Green real-time PCR assays in profiling the same two reference RNA samples analyzed by the MAQC Consortium. Using the MAQC data sets available from the public database [8,12,20], we conducted similar analyses for the RT^2 ^Profiler PCR Arrays and compared our expression profiling results with those generated from the three quantitative technologies (TaqMan, Sta*RT-*PCR and QuantiGene) as well as from five of the commercial microarray platforms (AFX, AG1, ABI, GEH and ILM) examined in the MAQC project [8,12].

## Results

### Assay Performance of SYBR Green Real-time PCR

To assess the performance of SYBR Green real-time PCR, the same two reference RNA samples of the identical manufacturing lots as the MAQC study [8,12] were analyzed using SYBR Green RT^2 ^Profiler PCR Arrays from SuperArray Bioscience. Sample A was Universal Human Reference RNA from Stratagene and Sample B was Human Brain Reference RNA from Ambion. In MAQC phase 1 study, 1297 genes were selected for cross-platform comparisons between microarrays and the quantitative platforms [8]. Among these 1297 genes, 997, 244 and 205 genes were assessed by TaqMan, QuantiGene and Sta*RT*-PCR assays, respectively. The gene list for the custom RT^2 ^Profiler PCR Array in this study was designed to overlap with the maximum possible number of genes for which MAQC data are available from the three quantitative platforms in the MAQC project (Table 1). A total of 90 genes were selected, among which 86 genes overlapped with TaqMan data, 76 genes overlapped with QuantiGene data, and 57 genes overlapped with Sta*RT*-PCR data. In addition, these 90 selected genes overlapped with data available for 89 genes on the AFX and ABI, 86 on the AG1, 88 on the GEH, and 87 on the ILM platforms. The custom PCR Array was run in six technical replicates for each of the two MAQC reference RNAs (Samples A and B). For cross-platform comparisons between SYBR Green PCR Arrays and other gene expression analysis platforms evaluated in the MAQC study, data from the other technologies were obtained from published results [8,12] and from the database accessible from the MAQC website [20]. The comparison of performance metrics between SYBR Green PCR Arrays and the other three quantitative platforms assessed in the MAQC study is summarized in Additional file 1.

**Table 1:** Gene list of the custom SYBR Green RT^2 ^Profiler PCR Array showing the overlaps with the other three quantitative gene analysis technologies

**Probes present in all three quantitative platforms**	**Gene Symbol**	**TaqMan and QuantiGene**	**Gene Symbol**	**TaqMan and Sta*RT*-PCR**	**Gene Symbol**
1	*ABCD2*	42	*ABCC2*	72	*ANXA1*
2	*ADM*	43	*ABCD1*	73	*ARHGDIB*
3	*AES*	44	*ABCG5*	74	*ATP1B2*
4	*ANXA5*	45	*AFP*	75	*CDK8*
5	*BAG1*	46	*APC*	76	*DAP *
6	*BRCA2*	47	*APOB*	77	*ERCC5*
7	*CCNA2*	48	*APOH*	78	*GSTP1*
8	*CDK9*	49	*BAG4*	79	*GSTT1*
9	*CDKN1A*	50	*CCL20*	80	*NTRK3*
10	*CYP1B1*	51	*CDK5R1*	81	*PCNA*
11	*DAD1*	52	*CHEK1*	82	*PPP3CA*
12	*DPP4*	53	*CHGA*	83	*PTCH *
13	*FADD*	54	*COL1A1*	84	*TP53*
14	*FGF9*	55	*CSNK2A2*		
15	*FOXA1*	56	*DRD5*	**House Keeping Genes**	**Gene Symbol**
16	FURIN	57	EDN1	85	B2M
17	*G6PD*	58	EGFR	86	*ACTB*
18	*ICAM1*	59	*EIF2AK2*	87	*GAPDH*
19	*IGF2R*	60	*ERBB4*	88	*HPRT1*
20	*IGFBP2*	61	*GFAP*	89	*POLR2A*
21	*IGFBP5*	62	*IGF2*	90	*RPL13A*
22	*IL8*	63	*IGFBP1*		
23	*INPPL1*	64	*JAK2*		
24	*JUN*	65	*JUNB*		
25	*KCNS3*	66	*KCNC1*		
26	*KDR*	67	*MIF*		
27	*KIT*	68	*MMP1*		
28	*LDLR*	69	*RAD51*		
29	*MAP2K6*	70	*STAT4*		
30	*MAP3K14*	71	*TFF1*		
31	*MX2*				
32	*MYB*				
33	*MYC*				
34	*PTGS2*				
35	*RAD52*				
36	*RARA*				
37	*RB1*				
38	*SELE*				
39	*SLC2A1*				
40	*SOD1*				
41	*TYMS*				

#### Assay precision

**Figure 1:**
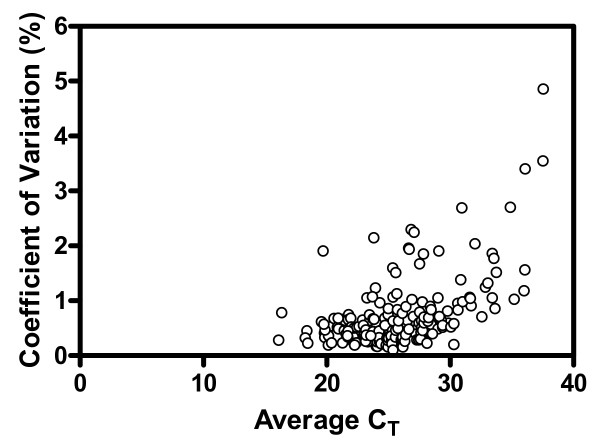
**Assay precision of SYBR Green RT^2 ^Profiler PCR Arrays.** The plot shows the coefficient of variation for each average C_T _value. A total of 184 average C_T _measurements were obtained from Sample A and Sample B. Each average C_T _value and its coefficient of variation were calculated from the result of 6 replicate assays.

The precision of the SuperArray's SYBR Green real-time PCR Arrays is assessed by the coefficient of variation (CV) and standard deviation (SD) of the replicate C_T _measurements (n = 6 for each assay) from both Samples A and B (Figure 1 and Table 2). The average CV for the 184 mean C_T _values generated from all assays on the custom PCR Arrays is 0.73%. All assays on PCR Arrays have an average C_T _value showing a CV below 5% with 95% of the C_T _values having a CV of less than 2% (Figure 1). The replicate measurements for C_T _values below 30 show an average standard deviation within 0.20 cycle (Table 2). As noted in Figure 1 and Table 2, both CV and SD tend to increase with increasing C_T _values (i.e. a decreasing amount of transcripts); this trend has also been observed for both TaqMan and Sta*RT*-PCR [8]. The above precision assessment also includes variations from the entire process of reverse transcription-PCR as each replicate array was performed on cDNA synthesized from individual replicate reverse transcription reactions. To make a comparison with the other three quantitative technologies examined in the MAQC project, the C_T _values obtained from PCR Arrays were transformed from a decreasing copy number scale to an increasing copy number scale applied in the MAQC study [8]. Using the same assumption as for TaqMan PCR where a C_T _value of 35 corresponds to five transcript molecules, the C_T _value for 6000 transcript molecules is extrapolated to be 24.78. This value is calculated to be 12.55 (log_2 _6000) on the common MAQC transformed log_2 _scale. After data transformation, the CVs for all 184 assays on PCR Arrays range from 0.18% to 74.36% with a median of 0.89%, and those for assays detecting > 6000 transcript copies (n = 70) range from 0.19% to 3.16% with a median of 0.57%.

**Table 2:** The average standard deviation for different C_T _value ranges and the percentage of genes in each group (percent frequency)

**C_T _Values**	**Ave SD**	**Frequency (%)**
10–25	0.11	45
25–30	0.19	41
30–35	0.40	11
≥ 35	0.96	3
Not Detectable	0	0

A total of 184 average C_T _measurements were obtained from Sample A and Sample B. Each average C_T _value and its standard deviation were calculated from the result of 6 replicate assays.

#### Detection sensitivity

PCR Array quantification is determined by C_T _numbers. A gene is considered absent when the average C_T _exceeds 35. Among the 90 genes that were assayed with SYBR Green PCR Arrays, seven genes are considered absent in sample B with the average C_T _> 35. These genes are *FOXA1*, *ABCC2*, *APOB*, *APOH*, *MMP1*, *RAD51 *and *TFF1*. All of these genes were considered to be either absent or expressed at low levels as measured by TaqMan and QuantiGene [8]. *FOXA1 *was also noted to be absent by Sta*RT*-PCR while the other six genes were not included in the measurements by this method. All genes are present in Sample A at a level above the limit of quantitation (referred to as the limit of detection (LOD) in the MAQC study [8]) of PCR Arrays with the average C_T _values smaller than 35. Hence, 83 out of the 90 selected genes (92%) are present in both Sample A and Sample B.

#### Assay range

The assay range is indicated by the difference in signals on a log_10 _scale between the highest and the lowest expression as described previously [8]. The assay range for the SYBR Green PCR Arrays is 8.6 with C_T _values ranging from 6.5 for 18S rRNA to 35 for weakly expressed genes.

#### Inter-site reproducibility

**Figure 2:**
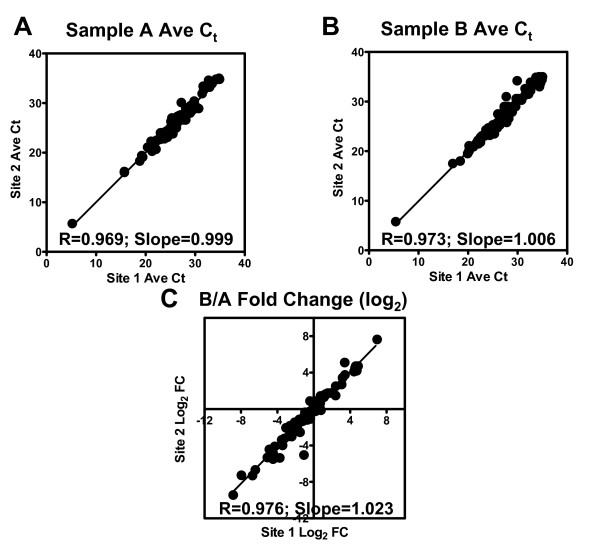
**Inter-site comparison of RT^2 ^Profiler PCR Arrays.** Threshold cycle measurements and fold-change results have been obtained using the two MAQC reference RNAs analyzed on the Human Drug Metabolism RT^2 ^Profiler PCR Array (APH-002) using different real-time PCR thermal cyclers at two different sites. Five replicate arrays were run for each sample at each site, and the average C_T _value for each assay is compared between the two sites in panels A and B. The results show a high correlation between C_T _values obtained from the two sites. Panel C shows the inter-site comparison of fold-change results obtained from these data, indicating a correlation coefficient of 0.976.

Inter-site comparison of the performance of SYBR Green PCR Arrays was carried out using the Human Drug Metabolism RT^2 ^Profiler™ PCR Array (Cat# APH-002) from SuperArray Bioscience. For inter-site comparison, the two MAQC reference RNAs were analyzed on the PCR Array at two different locations. PCR Arrays were performed on an ABI 7500 Real-Time PCR System at Site 1 while the arrays were run on an ABI 7000 at Site 2. Five replicate arrays were run for each sample at each site. The average C_T _value for each assay was compared between the two sites (Figure 2A and 2B). The results show a high correlation between C_T _values obtained from the two sites with R = 0.969 and 0.973 for Sample A and Sample B, respectively (Figure 2A and 2B). Moreover, the inter-site comparison of fold-change results obtained from these data shows a correlation coefficient of 0.976 (Figure 2C). Since the two test sites used two different models of real-time PCR thermal cyclers from Applied Biosystems for the above comparison, we investigated if the use of different real-time PCR instruments could contribute to the differences in the C_T _and fold-change results. We compared the gene expression results obtained using three different real-time PCR thermal cycler models from different manufacturers (ABI 7500, Stratagene Mx3000P™ and BioRad iCycler iQ™) and found that the correlations observed among different models of real-time PCR instruments are similar to those between the two test sites (data provided in Additional file 2).

### Cross-platform Comparisons with Other Technologies

To evaluate the concordance of fold changes between SYBR Green PCR Arrays and other technologies evaluated in the MAQC study, we performed regression analyses of fold differences in sample B compared to sample A for all genes common between the custom RT^2 ^Profiler PCR Arrays and another platform. In addition, a list of differentially expressed genes (DEGs) was identified for each platform between the two reference RNA samples using the cut-off criteria of a *P *value less than 0.05 by an unpaired *t*-test with a mean difference greater than or equal to 2-fold, and the lists of DEGs from different platforms were compared.

#### Fold-change correlation between PCR Arrays and other quantitative platforms

**Figure 3:**
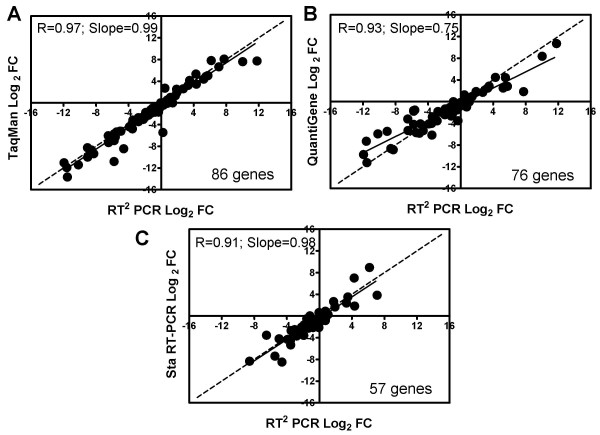
**Cross-platform comparison between SYBR Green RT^2 ^Profiler PCR Arrays and the three quantitative platforms.** The concordance of fold-changes between the PCR Arrays and the three quantitative platforms was evaluated by regression analysis of fold differences in sample B compared to sample A. Data were normalized against *POLR2A *for RT^2 ^Profiler PCR Arrays and TaqMan, and against beta-actin for Sta*RT*-PCR. The sample B/sample A (B/A) fold changes (log_2_) for each gene common between RT^2 ^Profiler PCR Arrays and another platform were subjected to bivariate analysis. The dashed line on each graph represents the ideal slope of 1.0. The solid lines show a linear regression fit.

SYBR Green RT^2 ^Profiler™ PCR Arrays display high concordance with TaqMan, QuantiGene and Sta*RT*-PCR in measuring fold differences between Sample A and Sample B. Data from regression analysis show that the correlation coefficient R and slope for RT^2 ^Profiler PCR Arrays versus TaqMan are 0.97 and 0.99, respectively; for RT^2 ^Profiler PCR Arrays versus QuantiGene are 0.93 and 0.75, respectively; and for RT^2 ^Profiler PCR Arrays versus Sta*RT*-PCR are 0.91 and 0.98, respectively (Figure 3 and Table 3). RT^2 ^Profiler PCR Arrays are shown to be best correlated with TaqMan assays (R = 0.97). When these results are compared with the MAQC data sets, similar fold-change correlations versus QuantiGene and Sta*RT*-PCR are noted for both RT^2 ^Profiler PCR Arrays and TaqMan (Table 3). Fold-change results between Sample A and Sample B from all four quantitative platforms and the lists of differentially expressed genes (DEGs) generated using the pre-set criteria (fold change ≥ 2, *P *< 0.05) are compared. PCR Arrays display a high percentage of cross-platform overlap with the other quantitative platforms, ranging from 81% to 93%.

**Table 3:** Fold-change correlation between the four different quantitative gene expression analysis platforms

**Correlation (R) **	**TaqMan**	**QuantiGene**	**Sta*RT*-PCR**
**(Number of genes in comparison)**			
**RT^2 ^Profiler PCR Array**	0.97 (86)	0.93 (76)	0.91 (57)
**TaqMan**		0.90* (181)	0.94* (92)
**QuantiGene**			0.92* (53)

*Correlation R values derived from the data provided by MAQC studies [8]

#### Fold-change correlation between PCR Arrays and microarray platforms

**Figure 4:**
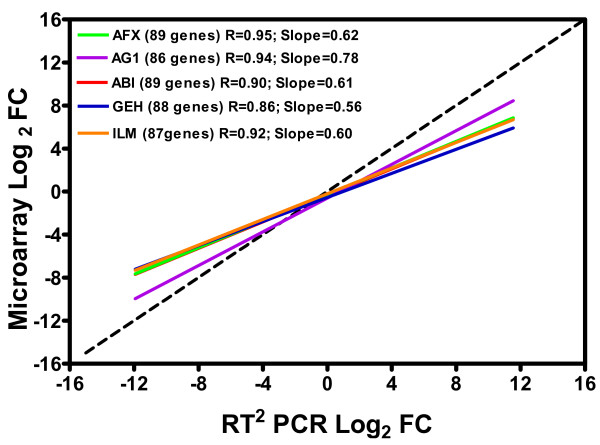
**Cross-platform comparison between SYBR Green RT^2 ^Profiler PCR Arrays and microarray platoforms.** The concordance of fold-changes between the PCR Arrays and microarray platforms was evaluated by regression analysis of fold differences in sample B compared to sample A. Data were normalized against *POLR2A *for RT^2 ^Profiler PCR Arrays. The sample B/sample A (B/A) fold changes (log_2_) for each gene common between RT^2 ^Profiler PCR Arrays and another platform were subjected to bivariate analysis. The dashed line on the graph represents the ideal slope of 1.0. Each solid line represents the linear regression fit. The numbers in brackets indicate the number of common genes between RT^2 ^Profiler PCR Arrays and each microarray platform. The individual scatter plot for each comparison is provided in Additional file 3.

Results from regression analysis showed a good linear correlation in the log_2 _fold change data between SYBR Green PCR Arrays and the five microarray platforms (AFX, AG1, ABI, GEH and ILM) selected for this study (Figure 4 and Table 4; see Additional file 3 for the individual scatter plot for each comparison). The correlation coefficients and linear slopes for the comparison between PCR Arrays and different microarray platforms are as follows: 0.95 and 0.62, respectively, for AFX; 0.94 and 0.78, respectively, for AG1; 0.90 and 0.61, respectively, for ABI; 0.86 and 0.56, respectively, for GEH; and 0.92 and 0.60, respectively, for ILM. The above results from these cross-platform comparisons with microarrays are very similar to those obtained from TaqMan as seen in the MAQC project (Table 4). The overlap in the lists of DEGs between PCR Arrays and each of the five microarrays is also high, yielding a 73% to 90% range.

**Table 4:** Fold-change correlations between SYBR Green RT^2 ^Profiler PCR Arrays or TaqMan assays and the five microarray platforms

**Correlation (R) **	**AFX**	**AG1**	**ABI**	**GEH**	**ILM**
**(Number of genes in comparison)**					
**PCR Array**	0.95 (89)	0.94 (86)	0.90 (89)	0.86 (88)	0.92 (87)
TaqMan*	0.92 (451–472)	0.91 (532–595)	0.85 (523–567)	0.85 (660–670)	0.91 (484–516)

*The average correlation R values and the number of genes in comparison for three sites are derived from the data provided by MAQC studies [12].

#### Discordant gene analysis

Comparisons between the fold-change results of the SYBR Green PCR Arrays and those of the other platforms reveal discordance in a few genes (Table 5). We define a gene to be discordant if the gene exhibits changes in the opposite direction with PCR Arrays and another platform being compared, and at least one of these two platforms indicates the gene to be differentially expressed (i.e. a twofold or greater change with a p-value less than 0.05). By these criteria, two genes, *FURIN *and *RB1*, are found discordant between PCR Arrays and the other quantitative platforms (Table 5). TaqMan shows a fold change of -43.98 for *FURIN *but PCR Arrays indicate a fold change of 1.16 for this gene. Sta*RT*-PCR, QuantiGene and the five microarray platforms show a fold change ranging from -1.49 and 1.04 for *FURIN*. Hence, no difference in the expression of *FURIN *between Samples A and B is observed with any platforms except TaqMan.

**Table 5:** Comparison of fold-change results on the discordant genes between SYBR Green PCR Arrays and other platforms

	**RT^2 ^Profiler PCR Array**	**TaqMan**	**Sta*RT*-PCR**	**QuantiGene**	**AFX**	**ABI**	**AG1**	**GEH**	**ILM**
***FURIN***	1.16	**-43.98**	-1.49	-1.42	-1.21	1.04	-1.38	N/A	-1.12
***RB1***	1.73*	-1.92	-1.59	**-3.09**	**-2.23**	-1.30	**-4.06**	**-2.14**	**-2.14**
***ABCD1***	-5.44	-7.11	N/A	-3.83	-1.45	**2.21**	-8.25	**2.15**	-2.41
***BAG1***	1.66	-1.06	1.85	-1.97	**-2.89**	-1.00	1.12	-1.01	1.18
***BAG4***	2.66	1.01	N/A	1.96	1.30	**-2.25**	**-2.25**	1.13	1.51
***CDK5R1***	45.53	28.92	N/A	21.55	18.11	**-1.09**	1.56	2.11	14.26
***IGFBP5***	-1.68	-2.69	-1.98	-2.16	-2.68	**4.11**	-3.26	-2.42	-2.37
***JAK2***	2.11	1.31	N/A	1.25	1.06	1.11	**-1.15**	1.99	1.06

Fold-change values for the above platforms other than RT^2 ^Profiler PCR Arrays are derived from the data provided by MAQC studies [8,12]. A positive value for fold change indicates a higher expression while a negative value indicates a lower expression in Sample B when compared with Sample A. "N/A" means the gene was not included in the indicated platform. Discordant fold-change values compared to RT^2 ^PCR Arrays are underlined and in bold. *The fold-change value for *RB1 *gene from the PCR Array using the corrected sequence is -1.75 (see text for details).

The other discordant gene, *RB1*, exhibits a fold change of 1.73 with PCR Arrays but a fold change of -3.09 with QuantiGene. TaqMan and Sta*RT*-PCR indicate fold changes of -1.92 and -1.59, respectively, for *RB1*. All of the five microarray platforms show negative fold changes with four of the platforms indicating a twofold or greater change. Interestingly, the *RB1 *gene was originally indicated by PCR Arrays to be absent (C_T _> 35) in both samples A and B. This finding was in contrast to the results from the other three quantitative platforms where the gene was considered to be present and moderately to highly expressed in both RNA samples [8]. This discrepancy was later found to arise from the probing location of the primers for *RB1 *in PCR Arrays being based on a former version of the RefSeq accession (NM 000321.1) which had been revised to the current RefSeq release (NM 000321.2) on June 9, 2006. The revised RefSeq accession revealed a two-base mismatch (from C-G to G-C) with the former version in the region where one of the *RB1 *primers for PCR Arrays happened to recognize. Upon repeating the assays using the *RB1 *primer with the revised sequence, the *RB1 *gene is now considered to be present with an average C_T _value of around 23–25 in both samples. Results generated from this new *RB1 *assay show a fold change of -1.75 for this gene, which is in agreement with TaqMan and Sta*RT*-PCR, and have replaced the data obtained from the old *RB1 *assay for the rest of the data analyses in this study.

Six other discordant genes besides *RB1 *are noted between SYBR Green PCR Arrays and the five microarray platforms (Table 5). Of those six discordant genes, one gene with AFX, four with ABI, two with AG1, and one with GEH, display changes in the opposite direction when compared to the RT^2 ^Profiler PCR Array. *BAG1 *exhibits a fold change of 1.66 with PCR Arrays but a fold change of -2.89 with AFX while the other platforms indicate the gene to be similarly expressed in both RNA samples with a minimal fold change from -1.97 to 1.85. *CDK5R1 *and *IGFBP5 *are shown to be discordant between ABI and PCR Arrays, with the directions of fold-change observed by ABI for these two genes to be opposite to those reported by all other platforms. *ABCD1 *and *BAG4 *are discordant within the five microarray platforms while the three quantitative platforms that have measured these two genes, the RT^2 ^Profiler PCR Array, TaqMan and QuantiGene, show fold-change values in a uniform direction. *JAK2 *displays a fold change of -1.15 by AG1 but 2.11 by PCR Arrays. All other platforms show the same direction of fold-change as PCR Arrays; however, like AG1, all of them indicate no significant difference in *JAK2 *expression between the two RNA samples with a fold change smaller than two.

## Discussion

Although SYBR Geen PCR is a popular gene expression technique used by about half of the real-time PCR users, it was surprisingly not evaluated in the MAQC study. Therefore, in this study, we have assessed the performance of SYBR Green RT^2 ^Profiler PCR Arrays and compared our expression profiling results of those two reference samples with the MAQC results obtained from three other traditional quantitative platforms including TaqMan assays, QuantiGene and Sta*RT*-PCR as well as five commercial microarrays.

The present study demonstrates SYBR Green RT^2 ^Profiler PCR Arrays to be a quantitative platform with high inter-run and inter-laboratory reproducibility. The average CV for the C_T _values generated from all assays on the custom PCR Array is found to be 0.73% with replicate measurements for C_T _values below 30 within 0.20 cycle average standard deviation, demonstrating a good inter-run reproducibility. When compared with the results from the other three quantitative platforms studied in the MAQC study (Additional file 1), the transformed data based on the corresponding universal MAQC scale give a median CV for all assays on PCR Arrays of 0.89% and that for assays detecting > 6000 transcript copies of 0.57%. These respective values were reported to be 3.46% and 2.42% for TaqMan, 6.26% and 3.82% for Sta*RT*-PCR, and 2.16% and 2.12% for QuantiGene [8]. As seen with TaqMan assays and Sta*RT-*PCR [8], a trend of increasing CV and SD is observed with increasing C_T _values (i.e. decreasing amount of transcripts) which is attributable to the stochastic nature of the sample loading of transcript molecules which influences the magnitude of CV [8]. High level of inter-site reproducibility is also demonstrated for SYBR Green PCR Arrays by inter-site comparison of C_T _values and fold-change results leading to correlation coefficients of 0.97 and 0.98, respectively.

Results obtained from SYBR Green PCR Arrays show that 83 out of the 90 selected genes (92%) are considered to be present in both samples A and B, which is similar to the values reported in the MAQC study for the detection sensitivity of the other three quantitative platforms [8]. Eighty-six percent (86%), 94% and 91% of the tested genes were above the respective LOD of the TaqMan assay (857 out of 997 tested genes), Sta*RT*-PCR (193 out of 205 tested genes) and QuantiGene (223 out of 244 tested genes) in both samples A and B [8]. The assay range of PCR Arrays is 8.6 on a log_10 _scale, which is comparable to the assay range of 8.1 for TaqMan assay reported in the MAQC project [8] and wider than those of the other two quantitative platforms in the MAQC study, with Sta*RT*-PCR and QuantiGene having an assay range of 6.8 and 4.1, respectively [8].

Cross-platform comparisons of gene profiling results of the two MAQC reference RNA samples illustrate a remarkably good correlation between SYBR Green RT^2 ^Profiler PCR Arrays and other technologies tested in the MAQC studies. Specifically, PCR Arrays and the other two PCR-based methods, TaqMan and Sta*RT*-PCR, exhibit an exceedingly high concordance with values for their correlation coefficients and linear slopes close to 1. This result demonstrates that these three methods report very similar fold changes in gene expression between the two MAQC samples. Although high correlation coefficients are observed between PCR Arrays and the hybridization-based techniques such as QuantiGene and the microarrays, the values for the linear slope are lower, ranging from 0.56 to 0.78. These results are consistent with a compression effect on log_2 _fold change computed from the hybridization-based techniques which was also observed when comparisons between TaqMan PCR and QuantiGene or microarray platforms were made in the MAQC studies [8,12].

SYBR Green real-time PCR Arrays demonstrate a good concordance in the differentially expressed gene list with the three quantitative technologies and five microarray platforms examined in the MAQC projects. Great overlaps in the list of DEGs are noted for PCR Arrays and TaqMan as well as the other quantitative platforms, ranging from 81% to 93%. The list of DEGs generated by PCR Arrays is also highly comparable with the five microarray platforms, with overlaps ranging from 73% to 90%. However, disparities in the expression results are observed for two genes between PCR Arrays and quantitative platforms, and for six genes in addition to *RB1 *between PCR Arrays and the five microarray platforms (Table 5). One possible cause for the disparities was experimentally investigated and found due to the accuracy of sequence information provided by the NCBI RefSeq database. As the design of the primers and probes for gene expression measurement is usually based on the gene sequence information from the RefSeq and non-RefSeq databases, any inaccuracies in those databases or the presence of previously unknown SNPs or splice variants, will potentially affect the accuracy of the assays. This scenario was clearly illustrated by the SYBR Green assay for *RB1 *gene in this study where the discovery of a two-base mismatch in one of the *RB1 *primers was made with the current RefSeq accession. Since the RefSeq database is frequently being updated to improve the accuracy of its gene sequence information, it is important to have the sequences of the primers and probes checked periodically against the most updated version of the RefSeq database in order to ensure the accuracy of the assays.

**Figure 5:**
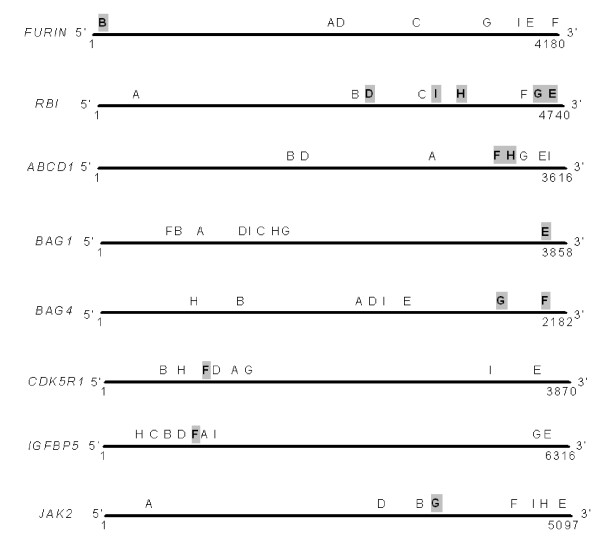
**Comparison of the probing locations for the discordant genes between RT^2 ^Profiler PCR Arrays and other gene expression measurement platforms in the MAQC phase 1 study.** The diagram illustrates the most 3' interrogative locations of the assays of different platforms for the eight discordant genes discussed in the text and shown in Table 6. The black lines represent, from left to right, the 5' to 3' position of the genes, each indicated with the length of the transcript. The assay location of each platform is indicated by a letter above the black line (A = RT^2 ^Profiler PCR Array; B = TaqMan; C = Sta*RT*-PCR; D = QuantiGene; E = AFX; F = ABI; G = AG1; H = GEH; and I = ILM). Assays with discordant results compared to those of PCR Arrays are highlighted in grey.

Because we have limited access to the information on the exact sequences of the primers and probes used by other technologies and platforms, it is difficult to investigate further all of the potential causes of the discordances between platforms by performing experiments. Another possible explanation for the discordance may be due to a difference in the interrogative regions [8]. The MAQC study provides the information on the approximate regions being probed in individual assays for all tested platforms in the form of the distance from the most 3' location of each assay to the 3' end of the gene [12]. Comparisons of the detection regions between different technologies are listed in Table 6 and shown in Figure 5. *FURIN *is found to be differentially expressed by TaqMan assays [8] but not by other platforms; this appears to be due to a difference in the regions being probed, with the TaqMan assay interrogating a region that is closer to the 5' end of the gene than those of other platforms [8] (Table 6 and Figure 5). The same explanation could also be suggested for *RB1*, *JAK2*, *BAG1 *and *BAG4*. For *RB1 *and *JAK2*, the locations being probed for PCR Arrays are closer to the 5' end of these genes than those of other platforms. Similarly, the discordance against other technologies in the fold-change results for *BAG1 *with AFX, and *BAG4 *with ABI and AG1, might be due to the fact that these platforms interrogate a region closer to the 3' end of these two genes than all other platforms.

**Table 6:** Comparison of the probing locations for the discordant genes between RT^2 ^Profiler PCR Arrays and other gene expression measurement systems in the MAQC study

	**Gene Length (Bases)**	**RT^2 ^Profiler PCR Array**	**TaqMan**	**Sta*RT-*PCR**	**QuantiGene**	**AFX**	**ABI**	**AG1**	**GEH**	**ILM**
***FURIN***	4180	2060	**4115**	1382	2048	65	1	632	N/A	184
***RB1***	4740	4268	2068	1742	**2053**	**46**	171	**119**	**1322**	**1471**
***ABCD1***	3616	1139	2317	N/A	2292	104	**565**	314	**355**	102
***BAG1***	3858	3143	3319	2675	2750	**148**	3381	2597	2614	2718
***BAG4***	2182	941	1618	N/A	924	584	**52**	**281**	1774	802
***CDK5R1***	3870	2823	3597	N/A	2905	41	**2948**	2782	3403	689
***IGFBP5***	6316	4845	5198	5519	5074	411	**4868**	418	5882	4740
***JAK2***	5097	4761	1409	N/A	2216	36	612	**1164**	316	323

The most 3' interrogative locations of the above assays are indicated by the distance to the 3' end of the corresponding gene. The probing locations of the assays for technologies other than RT^2 ^Profiler PCR Arrays are available from the supplementary information provided by the MAQC study [8,12]. Assays with discordant results compared to those of PCR Arrays are underlined and in bold.

The discrepancies in the expression results for the remaining three genes (*ABCD1*, *CDK5R1 *and *IGFBP5*) cannot simply be explained by the difference in probe locations. Interestingly, while these three genes display discordance among the microarray platforms, all four quantitative platforms show comparable results. This has led to the suggestion that disagreements between microarray platforms may be due to cross-hybridization of the probes on the arrays with other targets [8]. As in the case of *RB1*, the underlying reason for the discordance caused by different detection regions among technologies and platforms may be associated with the degree of accuracy in the sequence information being varied at different regions due to the continuous discoveries of previously unknown SNPs and spliced variants. Hence, even if the primers or probes from different platforms recognize the same region, there is a possibility that they may detect different spliced variants or transcripts with different SNPs. Again, frequently updating the primer and probe design with the most updated releases of RefSeq and non-RefSeq databases is essential for the success of all of these technologies.

Disagreements between different platforms may also arise from low detection signals for some genes. Among the discordant genes noted in Table 5, *BAG4 *and *CDK5R1 *are considered by all the five microarray platforms to be either absent or having relatively low expression in at least one of the two RNA samples. This trend with discordance occurring more frequently with low expressers was also noted in the MAQC study [8].

## Conclusion

In summary, SYBR Green real-time PCR Arrays produce gene profiling differences between the two MAQC reference RNA samples that are highly concordant with those generated by other quantitative gene expression analysis and microarray platforms. PCR Arrays deliver results comparable to those of high-density microarrays. Moreover, it yields results similar to those of TaqMan Gene Expression Assays, a widely accepted method for validating microarray results, and other more complicated and more expensive quantitative methods tested by the MAQC project. Hence, SYBR Green PCR Array is a quantitative platform suitable for microarray data validation.

## Methods

### Materials

Sample A (Universal Human Reference RNA) and Sample B (Human Brain Reference RNA), were purchased from Stratagene (Cat# 740000 lot#1130623; La Jolla, CA) and Ambion (Cat# 6050 lot#105P055201A; Austin, TX), respectively. Both of these reference RNA samples are from the identical manufacturing lots as those used in the MAQC study [8,12]. Reverse transcription kits (Cat# C-01) and SYBR Green real-time PCR master mixes (Cat# PA-012 and Cat# PA-011) were from SuperArray Bioscience (Frederick, MD). The Human Drug Metabolism RT^2 ^Profiler™ PCR Array (Cat# APH-002), that was used for inter-site and inter-instrument comparison, and a custom RT^2 ^Profiler PCR Array (Table 1), that was used for cross-platform comparison, were designed and manufactured at SuperArray with the primer sets for the specified genes pre-dispensed into a 96-well PCR plate.

### Cross-platform comparison

The custom PCR Array designed for cross-platform comparison was run in six technical replicates for each of the two MAQC reference RNAs. For each custom PCR array, one microgram (μg) of RNA was reverse transcribed in a 20-μL reaction volume into first-strand cDNA using SuperArray's ReactionReady™ First Strand cDNA Synthesis Kit (Cat# C-01) containing random primers following the instructions provided in the user's manual. After mixing the cDNA with RT^2 ^SYBR Green/ROX qPCR Master Mix (Cat# PA-012, SuperArray), 25 μL of the mixture containing cDNA synthesized from 9 ng total RNA was dispensed to each well of PCR Arrays. This amount of sample input is similar to the 10 ng total RNA sample input per reaction for TaqMan and Sta*RT*-PCR Assays but lower than the 500 ng total RNA input per reaction for QuantiGene performed in the MAQC phase 1 study. Real-time PCR was performed on an ABI 7500 Real-Time PCR System (Applied Biosystems, Foster City, CA) using the following cycling parameters: 10 min at 95°C (heat activation step); 40 cycles of 15 sec at 95°C, 1 min at 60°C. Dissociation curve analyses were performed using the instrument's default setting immediately after each PCR run.

### Inter-site comparison

The two MAQC reference RNAs were analyzed on the Human Drug Metabolism RT^2 ^Profiler™ PCR Array (Cat# APH-002) at two different locations with cDNA synthesis and PCR procedures performed as described above. PCR Arrays were performed on an ABI 7500 Real-Time PCR System at Site 1 while the arrays were run on an ABI 7000 at Site 2, with both sites using SuperArray's RT^2 ^SYBR Green/ROX qPCR Master Mix (Cat# PA-012). Five replicate arrays were run for each sample at each site.

### Data normalization and analyses

The threshold cycle number (C_T_) for each PCR reaction is determined by setting the same threshold value across all PCR Arrays. For data normalization in the custom PCR Arrays, *POLR2A *was selected as the reference endogenous control gene since it was used as the normalizer in the TaqMan Assay in the MAQC study [8]. *POLR2A *was measured on each array plate in triplicate assays. The comparative C_T _method was used to calculate relative quantification of gene expression as described previously [21]. The relative amount of transcripts for each gene in Sample A and Sample B was normalized to the reference gene *POLR2A *and calculated as follows: ΔC_T _is the log_2 _difference between the gene and the reference gene, and is obtained by subtracting the average C_T _of *POLR2A *from the C_T _value of the gene on a per array basis; the log_2 _fold change between the two samples was obtained using the formula: ΔΔC_T _= the average Δ C_T _of Sample B – the average Δ C_T _of Sample A, and their fold difference = 2^-ΔΔ ^C_T_. For cross-platform comparison, normalized data for both Sample A and Sample B from the other gene expression analysis technologies were directly obtained from published results [8,12] and from the database accessible from the MAQC website [20]. For each gene, the fold change between the two samples was generated by calculating the ratio of the average of the normalized signals for all sample B replicates to the average of the normalized signals for all sample A replicates. The sample B/sample A (B/A) fold changes (log_2_) for all genes common between the RT^2 ^Profiler PCR Arrays and another platform were compared and subjected to bivariate regression analysis, and Pearson correlation coefficients (R) were computed for each cross-platform comparison. For inter-site and cross-instrument comparison, data normalization in the Human Drug Metabolism RT^2 ^Profiler™ PCR Array was carried out as instructed in the user's manual using the mean C_T _of five housekeeping genes as the reference endogenous control for each array plate. The ΔΔC_T _and fold-change values between the two RNA samples for each gene on the array plate were obtained and compared between the two sites and across different real-time PCR thermal cyclers.

### Sensitivity detection and differentially expressed genes (DEG) determination

PCR Array quantification is determined by C_T _numbers. A gene is considered absent when the average C_T _exceeds 35. The C_T _is marked as 35 for the Δ C_T _calculation when the signal is below the limit of quantitation (this is referred to as the limit of detection (LOD) in the MAQC study [8]). A list of DEGs between the two reference RNA samples was identified using the cut-off criteria of a *P *value less than 0.05 as assessed by an unpaired *t*-test with a mean difference greater than or equal to 2-fold.

## Authors' contributions

EA designed and carried out the cross-platform comparative study, analyzed the results, and drafted the manuscript. YS and QZ prepared the materials for the experiments in this study. JW prepared the purified PCR products and carried out the standard curve experiments, and performed the experiments for the inter-site and inter-instrument comparative study. BN, SLD and LG participated in designing and carrying out the experiments for the inter-site comparative study. JY conceived the study, participated in its design and coordination and helped to draft the manuscript. All authors read and approved the final manuscript.

## Supplementary Material

Additional file 1 Comparison of performance metrics among the four quantitative platforms. The table shows the performance metrics of SYBR Green RT^2 ^Profiler PCR Array, TaqMan PCR, Sta*RT*-PCR and QuantiGene. Click here for file

Additional file 2 Correlations between real-time PCR instruments for the raw C_T _values and fold-change results between the two MAQC reference RNA samples analyzed on the Human Drug Metabolism RT^2^Profiler PCR Array (APH-002). The scatter plots show the correlation comparison among three different models of real-time PCR instruments for the raw C_T _and fold-change results generated from the two MAQC reference RNA samples on the Human Drug Metabolism RT^2^Profiler PCR Arrays. Click here for file

Additional file 3 The concordance of fold changes between SYBR Green-based RT^2^Profiler PCR Array and microarray platforms. The individual scatter plots for the comparison between the RT^2^Profiler PCR Array and each of the five microarrays are provided for the data presented in Figure 4 and Table 4 of the manuscript. Click here for file
