# Supplementary material for: Cross-platform comparison of SYBR® Green real-time PCR with TaqMan PCR, microarrays and other gene expression measurement technologies evaluated in the MicroArray Quality Control (MAQC) study
Source: BMC Genomics. 2008 Jul 11;9:328. doi: 10.1186/1471-2164-9-328 (PMC2491643; doi:10.1186/1471-2164-9-328)
Supplement: Additional file 1 — Comparison of performance metrics among the four quantitative platforms. The table shows the performance metrics of SYBR Green RT2 Profiler PCR Array, TaqMan PCR, StaRT-PCR and QuantiGene. [file 1471-2164-9-328-S1.ppt]

## Slide 1
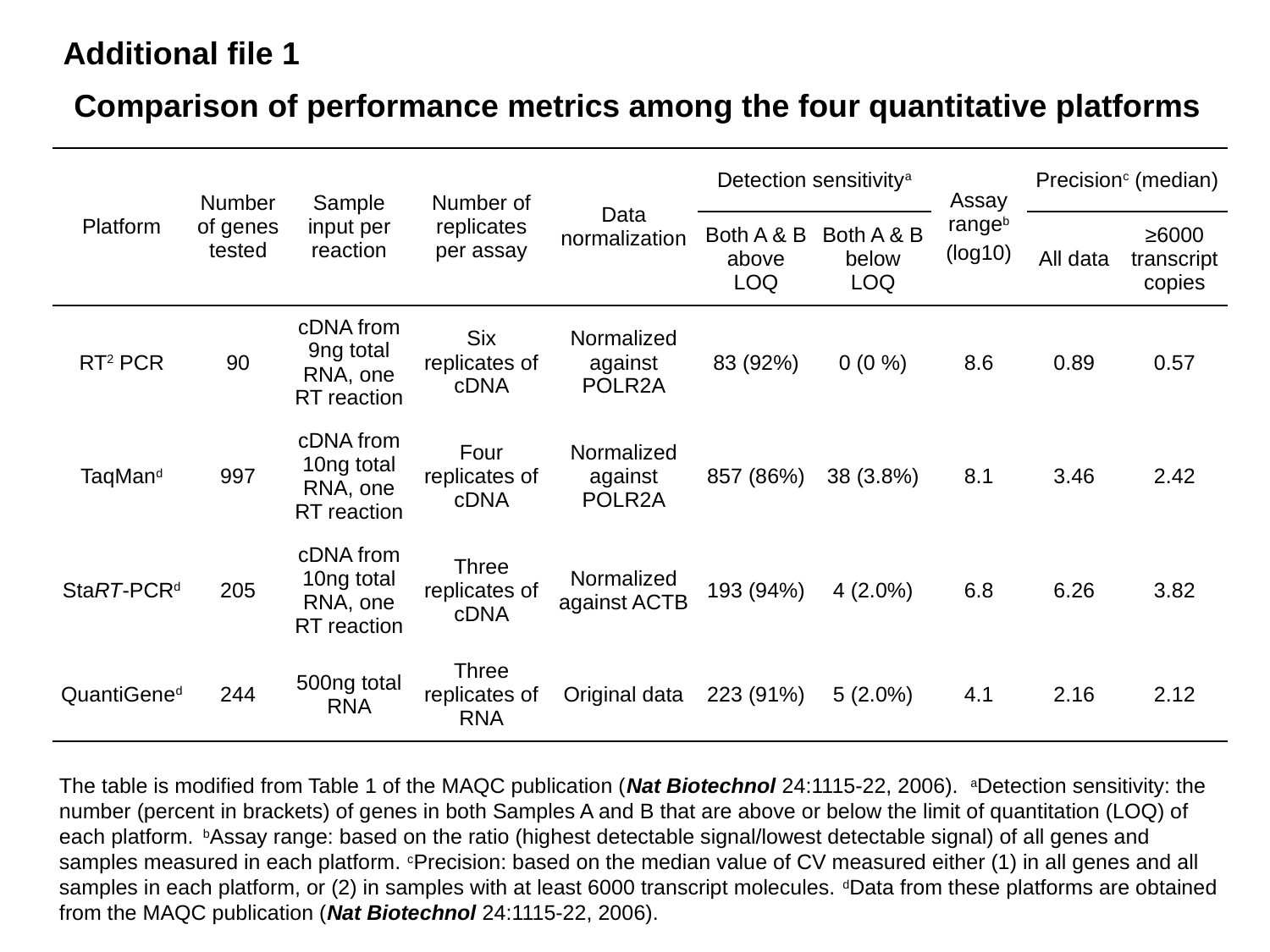

Additional file 1
Comparison of performance metrics among the four quantitative platforms
| Platform | Number of genes tested | Sample input per reaction | Number of replicates per assay | Data normalization | Detection sensitivitya | | Assay rangeb (log10) | Precisionc (median) | |
| --- | --- | --- | --- | --- | --- | --- | --- | --- | --- |
| | | | | | Both A & B above LOQ | Both A & B below LOQ | | All data | ≥6000 transcript copies |
| RT2 PCR | 90 | cDNA from 9ng total RNA, one RT reaction | Six replicates of cDNA | Normalized against POLR2A | 83 (92%) | 0 (0 %) | 8.6 | 0.89 | 0.57 |
| TaqMand | 997 | cDNA from 10ng total RNA, one RT reaction | Four replicates of cDNA | Normalized against POLR2A | 857 (86%) | 38 (3.8%) | 8.1 | 3.46 | 2.42 |
| StaRT-PCRd | 205 | cDNA from 10ng total RNA, one RT reaction | Three replicates of cDNA | Normalized against ACTB | 193 (94%) | 4 (2.0%) | 6.8 | 6.26 | 3.82 |
| QuantiGened | 244 | 500ng total RNA | Three replicates of RNA | Original data | 223 (91%) | 5 (2.0%) | 4.1 | 2.16 | 2.12 |
The table is modified from Table 1 of the MAQC publication (Nat Biotechnol 24:1115-22, 2006). aDetection sensitivity: the number (percent in brackets) of genes in both Samples A and B that are above or below the limit of quantitation (LOQ) of each platform. bAssay range: based on the ratio (highest detectable signal/lowest detectable signal) of all genes and samples measured in each platform. cPrecision: based on the median value of CV measured either (1) in all genes and all samples in each platform, or (2) in samples with at least 6000 transcript molecules. dData from these platforms are obtained from the MAQC publication (Nat Biotechnol 24:1115-22, 2006).
