# Supplementary material for: Cross-platform comparison of SYBR® Green real-time PCR with TaqMan PCR, microarrays and other gene expression measurement technologies evaluated in the MicroArray Quality Control (MAQC) study
Source: BMC Genomics. 2008 Jul 11;9:328. doi: 10.1186/1471-2164-9-328 (PMC2491643; doi:10.1186/1471-2164-9-328)
Supplement: Additional file 2 — Correlations between real-time PCR instruments for the raw CT values and fold-change results between the two MAQC reference RNA samples analyzed on the Human Drug Metabolism RT2Profiler PCR Array (APH-002). The scatter plots show the correlation comparison among three different models of real-time PCR instruments for the raw CT and fold-change results generated from the two MAQC reference RNA samples on the Human Drug Metabolism RT2Profiler PCR Arrays. [file 1471-2164-9-328-S2.ppt]

## Slide 1
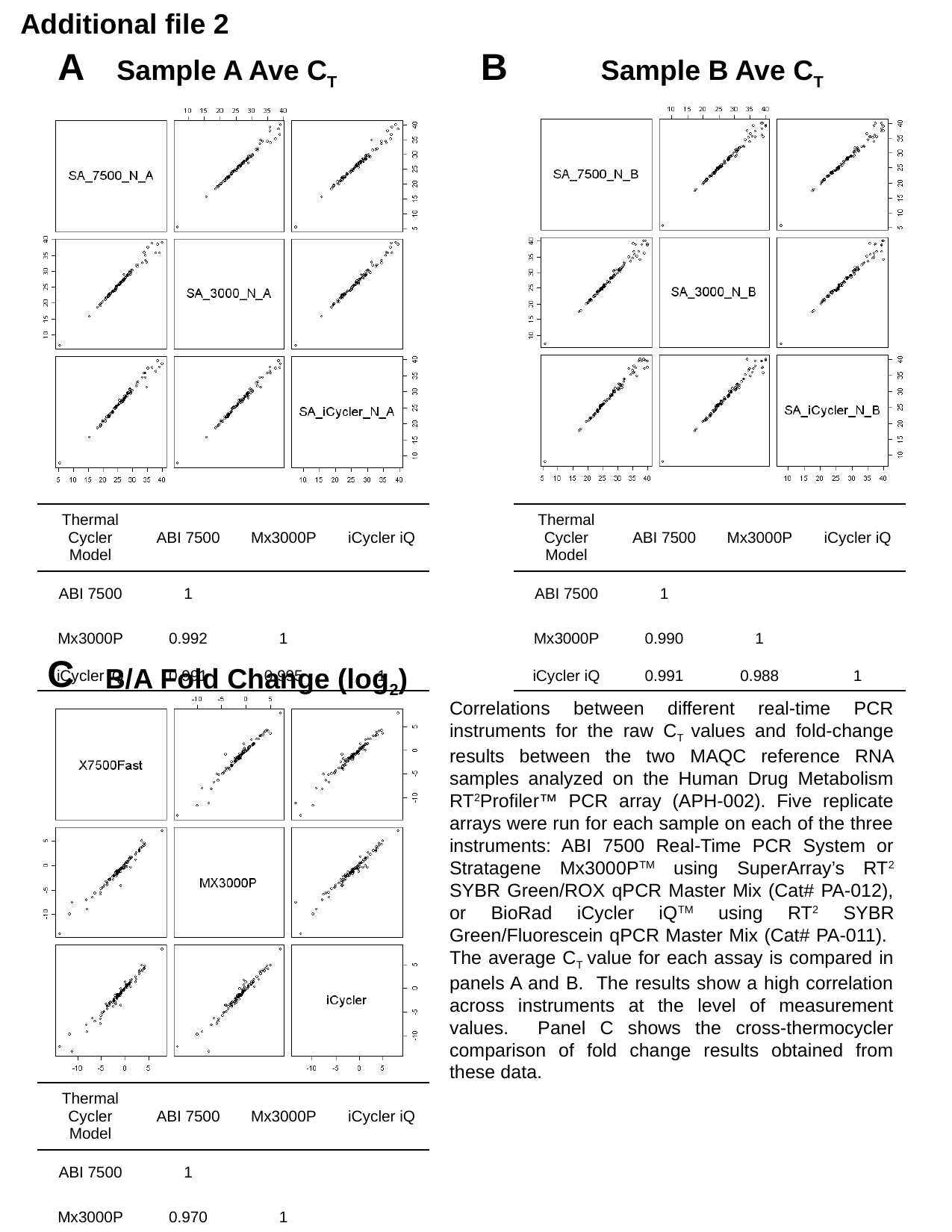

Additional file 2
A
B
Sample A Ave CT
Sample B Ave CT
| Thermal Cycler Model | ABI 7500 | Mx3000P | iCycler iQ |
| --- | --- | --- | --- |
| ABI 7500 | 1 | | |
| Mx3000P | 0.992 | 1 | |
| iCycler iQ | 0.991 | 0.995 | 1 |
| Thermal Cycler Model | ABI 7500 | Mx3000P | iCycler iQ |
| --- | --- | --- | --- |
| ABI 7500 | 1 | | |
| Mx3000P | 0.990 | 1 | |
| iCycler iQ | 0.991 | 0.988 | 1 |
C
 B/A Fold Change (log2)
Correlations between different real-time PCR instruments for the raw CT values and fold-change results between the two MAQC reference RNA samples analyzed on the Human Drug Metabolism RT2Profiler™ PCR array (APH-002). Five replicate arrays were run for each sample on each of the three instruments: ABI 7500 Real-Time PCR System or Stratagene Mx3000PTM using SuperArray’s RT2 SYBR Green/ROX qPCR Master Mix (Cat# PA-012), or BioRad iCycler iQTM using RT2 SYBR Green/Fluorescein qPCR Master Mix (Cat# PA-011). The average CT value for each assay is compared in panels A and B. The results show a high correlation across instruments at the level of measurement values. Panel C shows the cross-thermocycler comparison of fold change results obtained from these data.
| Thermal Cycler Model | ABI 7500 | Mx3000P | iCycler iQ |
| --- | --- | --- | --- |
| ABI 7500 | 1 | | |
| Mx3000P | 0.970 | 1 | |
| iCycler iQ | 0.998 | 0.976 | 1 |
