# Supplementary material for: Cross-platform comparison of SYBR® Green real-time PCR with TaqMan PCR, microarrays and other gene expression measurement technologies evaluated in the MicroArray Quality Control (MAQC) study
Source: BMC Genomics. 2008 Jul 11;9:328. doi: 10.1186/1471-2164-9-328 (PMC2491643; doi:10.1186/1471-2164-9-328)
Supplement: Additional file 3 — The concordance of fold changes between SYBR Green-based RT2Profiler PCR Array and microarray platforms. The individual scatter plots for the comparison between the RT2Profiler PCR Array and each of the five microarrays are provided for the data presented in Figure 4 and Table 4 of the manuscript. [file 1471-2164-9-328-S3.ppt]

## Slide 1
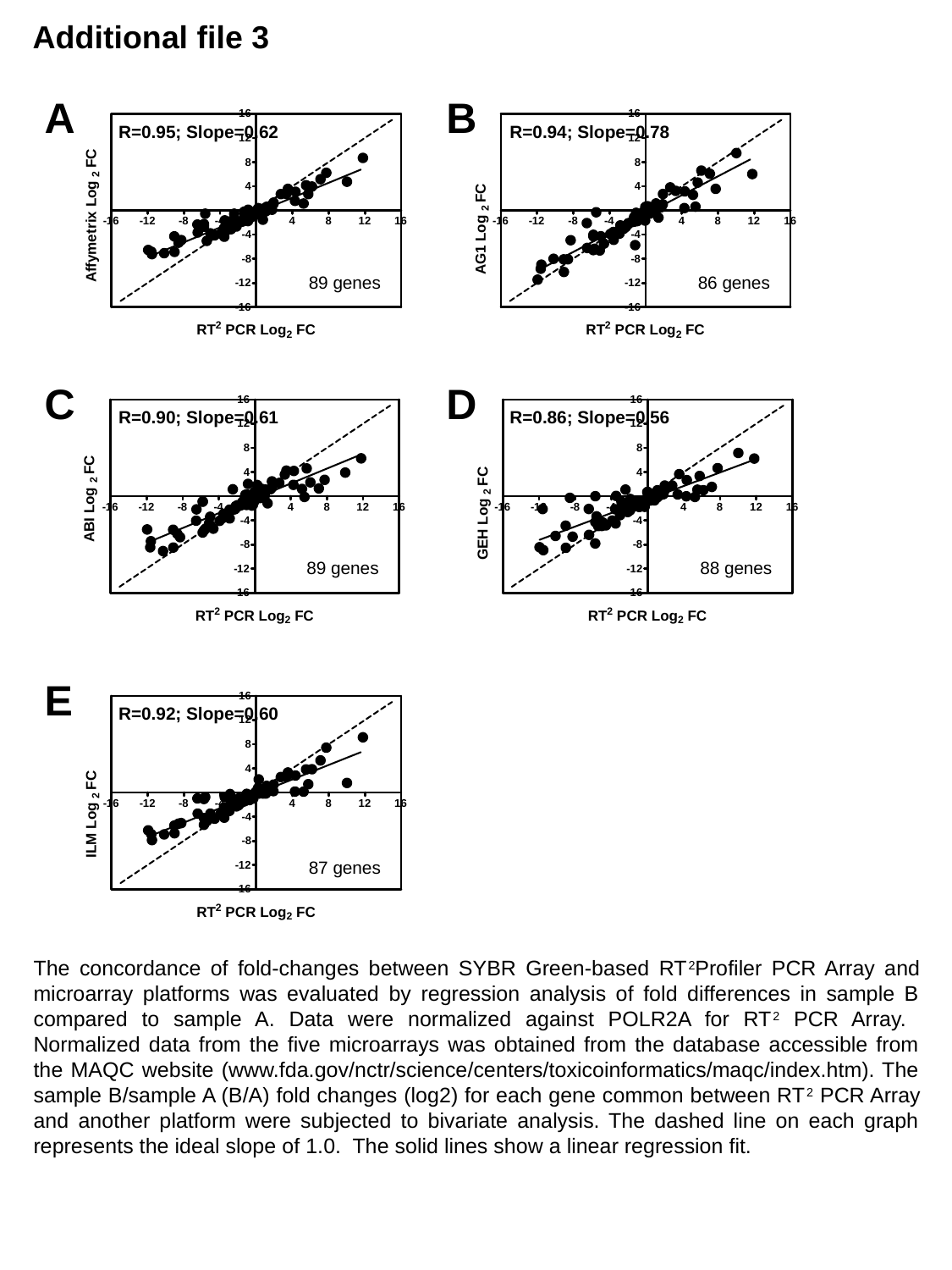

Additional file 3
A
B
89 genes
86 genes
R=0.95; Slope=0.62
R=0.94; Slope=0.78
C
D
89 genes
88 genes
R=0.90; Slope=0.61
R=0.86; Slope=0.56
E
87 genes
R=0.92; Slope=0.60
The concordance of fold-changes between SYBR Green-based RT2Profiler PCR Array and microarray platforms was evaluated by regression analysis of fold differences in sample B compared to sample A. Data were normalized against POLR2A for RT2 PCR Array. Normalized data from the five microarrays was obtained from the database accessible from the MAQC website (www.fda.gov/nctr/science/centers/toxicoinformatics/maqc/index.htm). The sample B/sample A (B/A) fold changes (log2) for each gene common between RT2 PCR Array and another platform were subjected to bivariate analysis. The dashed line on each graph represents the ideal slope of 1.0. The solid lines show a linear regression fit.
